# Comparison of effects of extra-thoracic paraaortic counterpulsation to intraaortic balloon pump on circulatory support in acute heart failure

**DOI:** 10.1186/s13019-015-0349-z

**Published:** 2015-11-24

**Authors:** Jie-Min Zhang, Xiao-Cheng Liu, Zhi-Gang Liu, Long Zhao, Li Yang, Tian-Wen Liu, Guo-Wei He

**Affiliations:** 1 Center for Circulatory Support and Animal Lab., & Center for Basic Medical Research, TEDA International Cardiovascular Hospital, No.61, the 3rd, TEDA, Tianjin, 300457 China; 2 Department of Surgery, Oregon Health and Science University, Portland, OR USA

**Keywords:** Heart failure, Ventricular assist device

## Abstract

**Background:**

Previously designed intra-thoracic paraaortic counterpulsation device has limited stroke volume and may depress the lung to cause complications. The purpose of this study was to evaluate the hemodynamic effects of an extra-thoracic paraaortic counterpulsation device (ETPACD) in comparison to intraaortic balloon pump (IABP) in an animal model with acute heart failure.

**Methods:**

The acute heart failure model was successfully induced by snaring branch of anterior descending coronary artery in sheep (weighting, 38-50 kg, *n* = 8). The ETPACD is a single port, 65-ml stroke volume blood chamber designed to be connected to descending aorta through a valveless graft and placed extra-thorax. In comparison, a standard clinical 40-ml IABP was placed in the descending aorta. The hemodynamic indices of both devices were recorded during counterpulsation assistance. Two of the sheep were allowed to survive for 1 week to examine the prolonged effect.

**Results:**

Both ETPACD and IABP increased cardiac output with higher effect of ETPACD (13.52 % vs. 8.19 % in IABP, *P* < 0.05) and on mean diastolic aortic pressure (26.73 % vs. 12.58 % in IABP, *P* < 0.01). Both ETPACD and IABP also produced a greater reduction in left ventricular end-diastolic pressure (26.77 % vs. 23.08 %, *P* > 0.05). The ETPACD increased left carotid artery flow more significantly the IABP (18.00 % vs. 9.19 % , *P* < 0.05). In two of the sheep allowed to survive for 1 week, the device worked well with no complications and there was no thrombus formation in the chamber of ETPACD.

**Conclusions:**

This study demonstrated that both ETPACD and IABP provided benefit of circulatory support in acute heart failure with better effect on hemodynamic parameters provided by ETPACD. Therefore, ETPACD with theoretical larger stroke volume may become a promising counterpulsation device for treatment of heart failure.

## Background

The incidence of congestive heart failure (CHF) is increasing worldwide, with more than one million new cases diagnosed annually. Current treatment options for CHF include optimal medical management therapy, mechanical ventricular assist, and heart transplantation. Over the past four decades, the intra-aortic balloon pump (IABP) is the most widely used short-term cardiac assist device with over 160,000 balloons implanted each year worldwide with up to 65 % successful clinical outcomes [1–3]. Despite notable advantages, including wide availability, percutaneous insertion, acceptable cost, and proven efficacy, the long-term use of the IABP to support patients with end-stage heart failure were seriously limited by vascular complications [4, 5], complete immobilization of the patient, a high incidence of poor results in presence of particularly severe cardiogenic shock and very low aortic pressures. The paraaortic counterpulsation device (PACD) has been proposed as an alternative for mechanical support of severe heart failure in patients [6]. It is a pneumatically driven, double or half-round, polyurethane chamber with a valveless orifice connected to the ascending or descending aorta through a Dacron vascular graft. This device, with counterpulsation volumes from 20 to 100 ml, was found superior to the IABP and effective even at very low levels of aortic pressure in short-term experiments [7–12]. In addition, the long-term efficacy and high biocompatibility of the PACD observed in chronic experiments [12] have been verified clinically [8]. However, main obstacles remain in the widespread clinical use of the PACD such as the complications of implantation and difficulties in closure of patient’s thoracic cavity.

The present study was designed to compare the hemodynamic efficacy of a 65-ml stroke volume ETPACD to the 40-ml IABP in an acute heart failure model and to explore feasible surgical procedures in placing extracorporeal (extra-thoracic) ETPACD in order to facilitate the closure of the chest and to avoid the complications in placing the device in an intra-thoracic location.

## Materials and methods

**Fig. 1:**
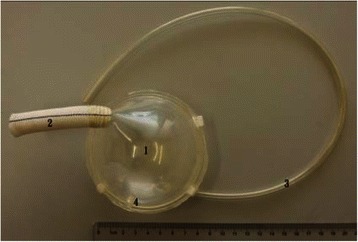
Photograph of an extra-thorax paraaortic counterpulsation device (ETPACD). The ETPACD sac fills with 120-ml (a 65-ml stroke) blood volume (1) during ventricular systole and empties during ventricular diastole through a single valveless cannula (2). The diaphragm is pneumatically driven by the console through a gas line (3) and the gas outlet (4)

**Fig. 2:**
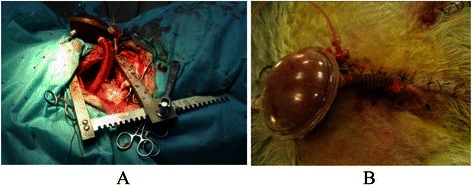
Photograph of experimental setup for the 65-ml extra-thoracic paraaortic counterpulsation device (ETPACD) in the acute heart failure sheep model. **a**. The vascular graft is anastomosed to the descending aorta and the device is left extracorporeal location with the thorax open; **b**. View of ETPACD after closure of the chest

A new extra-thoracic paraaortic counterpulsation device (ETPACD) (Fig. 1) (made in Dong Guan Ke Wei Medical Apparatus Co. Ltd., Title: A Paraaortic Counterpulsation Device . Patent Number: ZL 200920095633.X), the core component of the device is a 65-ml stroke volume polyurethane blood chamber, connected to the aorta by a valveless graft (Fig. 2). A drive line runs from the other chamber to an pneumatic driver. During systole, the drive console evacuates (helium) gas from the chamber thus removing blood from the aorta reducing cardiac work and afterload. During diastole, the console ejects blood from the blood chamber of the device into the aorta to provide augmentation of diastolic pressure and to improve coronary perfusion. The blood chamber has been specifically developed for continuous washing and nominal shear stress. The shape of the ETPACD is designed to double half-ball and wash continuously by creating a constant but shifting circular vortex of blood to minimize risk of thrombosis. The graft of the device connects seamlessly to the chamber without an inlet or outlet valve and supported with reinforcement rings. The distance between the chamber and descending aorta is minimized to reduce the risk of graft kinking and to improve efficiency.

**Fig. 3:**
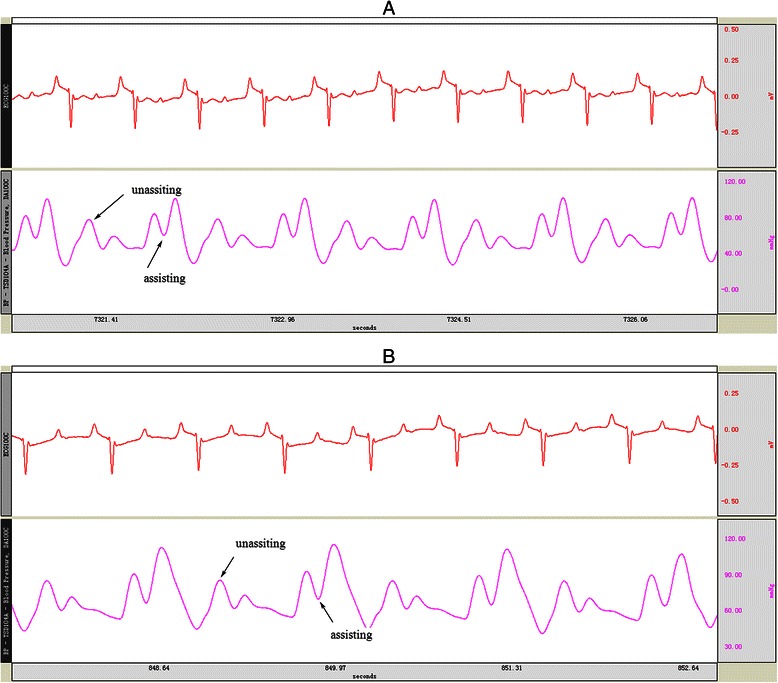
Actual trace of **a**. Aortic pressure wave with IABP assisting (1:2) and **b**. Aortic pressure wave with ETPACD assisting (1:2)

**Fig. 4:**
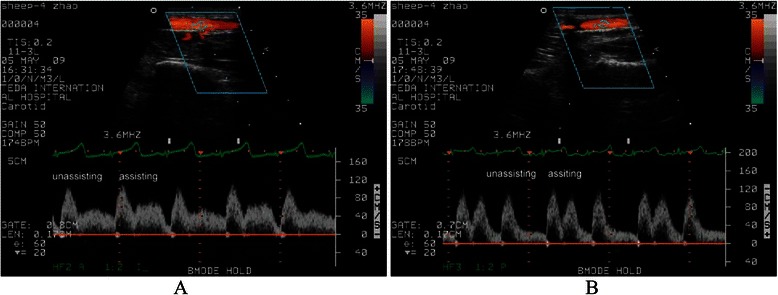
The left carotid artery flow with IABP (1:2) (**a**) and with ETPACD (1:2) (**b**)

### Animal preparations

The acute heart failure model was successfully established by snaring branch of descending coronary artery in eight healthy adult sheep (weighting 38-50 kg). All animals were offered standard humane care in compliance with the “Guide for the Care and Use of Laboratory Animals,” published by the National Institutes of Health (NIH publication 85–23, revised 1983). Ethical consent was approved by Ethics Committee of Laboratory Animals of TEDA International Cardiovascular Hospital. Food and water were withheld for 48, 24 h before the operation. The animals were premedicated with ketamine hydrochloride, 3 mg/kg (Gutian Pharmaceutical CO., Ltd, Fujian Province, China) midazolam, 0.1 mg/kg (Enhua Pharmaceutical CO., Ltd, Jiangsu Province, China), and anesthesia was induced with Propofol, 0.5 ml/kg i.v. (Libang Pharmaceutical CO., Ltd, Xian, China) administered as an slow i.v. injection. The trachea was intubated with a 7.5-mm cuffed endotracheal tube, followed by suxamethonium chloride, 1.0 mg/kg i.v. (Xudong Haipu Pharmaceutical CO., Ltd, Shanghai, China) for analgesia and neuromuscular blockade with ethrane (1 %–3 %) and fentanyl, 0.03 mg/kg i.v., respectively. Ventilation was controlled by intermittent positive pressure to maintain a PaO_2_ above 100 mmHg and the end-tidal CO_2_ between 30 and 40 mmHg. Anesthesia and analgesia were maintained by continuous intravenous infusions of Propofol 0.15 ml/kg/h and fentanyl 0.03 mg/kg/h. Neuromuscular blockade was maintained by a continuous intravenous infusion of suxamethonium chloride.

### Surgical procedures

The right external jugular vein was surgically exposed and cannulated with a 7.5 F Swan-Ganz catheter (Edwards Lifesciences LLC, USA ) to monitor the right atrial, right ventricle, and pulmonary arterial pressure, and to administer fluids. The left lateral thoracotomy was performed through the 4th intercostal space. Heparin 1 mg/kg was given by i.v. and ACT was maintained above 200 s. The descending aorta was partially clamped by a Satinski clamp and the Dacron vascular graft of ETPACD with the orifice of 12 mm in diameter was anastomosed end-to-side to the descending aorta. The ETPACD used in this study was the blood chamber, separated from the gas chamber by a movable polyurethane membrane (Fig. 1). It had a pumping 65-ml stroke volume when fully inflated. The gas conduit was directly connected to the driving line of the console. A 4 F multipurpose catheter was inserted through the left ventricular apex to record the left ventricular pressure. The experimental preparation was completed with the insertion of a standard 40-ml intraaortic balloon into the descending aorta via the surgically exposed left femoral artery . Baseline hemodynamic measurements were made before acute heart failure.

### Left ventricular failure model

After the pericardial sac was opened, left ventricular failure was induced by ligation of the left descending coronary artery and/or circumflex coronary arterial branches, starting from the peripheral segments of the left anterior descending coronary artery and lidocaine (2 mg/kg/h) was administered intravenously to prevent ventricular arrhythmias. The heart rate (HR), systolic aortic pressure (SAP), the mean aortic diastolic pressure (MADP), the central venous pressure (CVP), and the cardiac output (CO) were continuously monitored. After the acute heart failure model was successfully established, baseline hemodynamic measurements were made without mechanical support. The chest incision was then closed by layers around the vascular graft that was fixed by a short plastic tube to avoid kinking of the graft.

After the surgical procedure, the mechanical support was randomly initiated either by the IABP or by the ETPACD in each experiment. Both devices were driven by the same console (Datascope 98, Datascope Corp., Montvale, NJ, USA) and synchronization with the electrocardiogram (ECG) was used to provide air chamber inflation during diastole. Hemodynamic indices and the left carotid artery flow (LCAF) were recorded or calculated during 1) baseline (device off) and 2) 1:2 support for the 65-ml OTPACD or 40-ml IABP in the same animal. Baseline measurements were repeated between test conditions to ensure that CHF baseline values were maintained at a comparably steady state. In each experiment, the baselines and support mode for each device were maintained for 15 min to ensure a steady-state was reached. Data were collected for 60 s at the end of each steady-state test condition. The test order of devices (IABP and ETPACD) and support modes (1:2) were randomized to eliminate experimental order bias.

### Free hemoglobin and necropsy

Venous blood samples were drawn to measure total hemoglobin and plasma free hemoglobin for each experimental test condition and device trial. At the completion of the experiment, the blood chamber and graft of ETPACD were removed and visually inspected for gross evidence of thrombus, fractures, and other defects. Peripheral organs were removed and microscopical slides were prepared to examine thrombus related infarcts.

### Postoperative survival

**Fig. 5:**
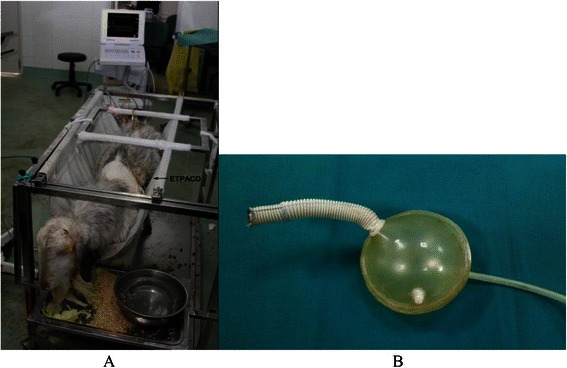
**a**: Photograph of a sheep one week after the implantation of extra-thoracic paraaortic counterpulsation device (ETPACD).The picture shows that the sheep survived well with the ETPACD (shown by the arrow). terpulsation device (ETPACD).  The picture shows that the sheep survived well with the ETPACD (shown by the arrow). **b**. The sheep was then sacrificed and the ETPACD was examined for possible thrombus formation. There was no thrombus formation in the chamber of ETPACD

In two of the sheep that had ETPACD, the ETPACD was continued to postoperative period with the continuation of the ETPACD support for 1 week (see Fig. 5a) with no major complications. The anti-coagulant therapy was continued by using heparin (1.0-2.0 mg/h) for 48 h to maintain ACT at 150–180 s and followed by oral warfarin (4.5-7.5 mg/day, dissolved in drinking water) afterwards to maintain INR at 1.5-2.0.

### Statistical analysis

BIOPAC’s AcqKnowledge 3.9 was used for post-acquixsition processing of hemodynamic data. The hemodynamic data acquired during the ETPACD- or IABP-assisted condition were compared with data acquired before the device-assistance. Data are presented as mean ± standard deviation. When the comparisons involved only two groups, the Student’s *t*-test for paired observations was used. The multiple comparisons among three groups were performed by One-Way ANOVA test with SPSS 13.0. A *p* value < 0.05 was considered statistically significant.

## Results

The acute heart failure model was successfully established in eight animals. The hemodynamic parameters before and after acute heart failure are shown in Table 1.

**Table 1:** Hemodynamic data before and after acute heart failure

	CO (L/min)	PCWP (mmHg)	MAP (mmHg)	LVEDP (mmHg)
Normal	3.84 ± 0.21	6.50 ± 1.06	93.75 ± 4.06	-1.00 ± 1.69
HF	2.75 ± 0.19*	11.00 ± 1.31*	59.13 ± 5.54*	3.25 ± 1.39*

*HF* heart failure, *CO* Cardiac output, *PCWP* pulmonary capillary wedge pressure, *MAP* mean aortic pressure, *LVEDP* left ventricular end-diastolic presssure Values are given as mean ± SD

^*^ Normal *vs*. HF (*p* <0.001)

Complete hemodynamic data before and during mechanical support by the IABP or ETPACD are shown in Table 2 and Fig. 3). Both IABP and ETPACD improved hemodynamic indices. However, the improvement by ETPACD was significantly greater than that by IABP. The left carotid artery flow was improved by 9.2 % in IABP (*P* <0.01) and 18.0 % in ETPACD (*P* <0.05) with more improvement by ETPACD, compared to IABP, *P* <0.05 (Fig. 4).

**Table 2:** Hemodynamic variables before and during mechanical support by the IABP or ETPACD in acute heart failure

Device	CO (L/min)	SAP (mmHg)	MADP (mmHg)	LVEDP (mmHg)	LCAF (ml/min)
HF baseline	2.81 ± 0.22	78.00 ± 5.81	55.63 ± 5.98	3.25 ± 1.39	234.75 ± 20.30
40-ml IABP	3.04 ± 0.20**	72.75 ± 5.80*	62.63 ± 7.37*	2.50 ± 1.31	258.50 ± 20.13**
65-ml OTPACD	3.19 ± 0.19*^,^***	66.13 ± 5.92*^,^****	70.50 ± 7.50*^,^****	2.38 ± 1.19	277.00 ± 19.00*^,^***

Values are given as mean ± SD. HF vs. IABP, HF vs. ETPACD (* *p* value < 0.05, **p value < 0.01)

IABP vs.OTPACD (*** *p* value < 0.05,**** *p* value < 0.01)

*HF* heart failure, *CO* cardiac output, *SAP* systolic aortic pressure, *MADP* mean aortic diastolic pressure, *LVEDP* left ventricular end diastolic presssure, *LCAF* left carotid artery flow

The plasma free hemoglobin levels remained 14-37 mg/L for the whole experiments. At the completion of the experiment, the blood chamber and graft of ETPACD were removed and there was neither gross evidence of thrombus, fractures, and other defects nor thrombus related infarcts.

In two of the sheep that had the ETPACD continuously used postoperatively for 1 week (Fig. 5), the device worked well with no complications. One week later, the sheep was sacrificed and the ETPACD was examined for possible thrombus formation. Neither of these sheep had thrombus formation in the chamber of ETPACD (Fig. 5b).

## Discussion

In the present study, we compared the efficiency of ETPACD with 65 ml stroke volume to that of IABP and we have found that 1) in treatment of acute heart failure, this device has higher efficiency than IABP also both work well and 2). The extra-thoracic location of the device is surgically feasible at least in the acute condition.

As mentioned above, heart failure is a major public health problem in the world, associated with high morbidity and mortality, estimated to impact over 20 million people worldwide. **I**ntra-aortic balloon pump (IABP) is a commonly used form of circulatory support, often for cardiogenic shock or severe left ventricular dysfunction following acute myocardial infarction or cardiotomy [13, 3] with acceptable survival (65 %) [2]. IABP worked to reduce afterload, increased diastolic aortic pressure, and increased coronary perfusion, in addition to promoting a slight increase in cardiac output. However, application of IABP is associated with a wide variety of complications, especially long-term assisting, the most common being bleeding, systemic embolization, limb ischemia, amputation and infection. IABP may also be associated with mechanical failure, such as balloon rupture, inadequate inflation, or inadequate diastolic augmentation. Particularly, IABP-related vascular complications have been reported in multiple studies, with an incidence of 8.7 % to 33 % [14–16].

On the other hand, although PACD was suggested to be a useful method to treat heart failure a few decades ago, its clinical use has been very limited and only few clinical reports on the use of PACD [8]. Reasons accounting for its limited use include depression of the thoracic organs, especially the lung that results in atelectasis and intrapulmonary infection leading to pulmonary dysfunction. Another problem is when the device is placed intra-thorax, the counterpulsation sac may be depressed resulting reduced function. In addition, one of the benefits of PACD is that its stroke volume is larger than IABP. The current experiments used a stroke volume of 65 cc with possibility to increase it to 80 cc or even more. This is obviously beneficial to the superior hemodynamic effect over IABP. However, with the quite large size of the device, when it is placed in the chest, it not only depresses the lung but also, may cause difficulties to close the chest.

Based on these considerations, we have designed a new extra-thorax paraaortic counpepulsation device. The present study has demonstrated the efficiency of this device. Indeed, we have shown that the hemodynamic improvement of this device is greater than IABP. By using ETPACD, the possible vascular complications of implantation of IABP is avoided. In addition, leaving the device extra-thorax, the ETPACD does not depress the lung and the closure of the chest become no problem. This gives a possibility in the future to increase the volume of the chamber and to individualize the size of the device according to the severity of the heart failure and the patient size.

Should the device be used for long-term, the vascular graft could be placed through another intercostal space to avoid the major incision problems. In this study, the fact that in two of the sheep that had the ETPACD continuously used postoperatively for 1 week, the device worked well with no complications and no thrombus formation in the chamber of ETPACD, indicating that this device has potential to be used for mid-term or long-term use postoperatively.

Furthermore, for a long-term use, the device could be easily observed for the function of the helium gas chamber as well as the blood chamber.

The valveless characteristics of the ETPACD consisting of polyurethane and heparin-coated inner surface make it highly biocompatible in order to reduce the use of anticoagulant agents and to decrease free hemoglobin in plasma, therefore suitable for long-term support of patients with heart failure. In addition, the implantation of the device allows mobilization of the patient, limited only by the driving line and console.

Lastly, the ETPACD was designed to be easily removed should the heart function be recovered and the use of ETPACD be terminated. A small thoracotomy is enough to facilitate the placing of large titanium clips near the aortic side of the vascular to completely close the graft.

### Clinical implications

Although the present study is an experimental investigation, we may draw some outlines for the possible indications to insert ETPACD. First, the indication for ETPACD is similar to IABP. When a patient has left ventricular failure after cardiac surgery with difficulty to come off cardiopulmonary bypass or need left ventricular support with low cardiac output, insertion of IABP or ETPACD could be considered. However, if it is predicted that IABP may not provide enough support such as in patients with low blood pressure in whom IABP may not provide high enough blood pressure for life support or an IABP has been inserted but not provide effective support, ETPACD may provide better support. Second, when an IABP support is used for more than a week or predicted to be lasting for more than a week, ETPACD may be considered since it has fewer vascular complications. Third, when the left ventricular failure is quite severe that may need left ventricular assistance device, ETPACD may be tried to insert as the first-line support. However, the above implications need to be further studied.

### Limitations

The efficacy of the PACD, however, is limited by the usual restrictions of counterpulsation techniques, including the requirement of a minimum aortic systolic pressure and stroke volume generated by the failing heart, a competent aortic valve to avoid regurgitation of the counterpulsation volume into the left ventricle thus creating unfavorable overload, as well as the constraints imposed by the heart rate, because excessively short diastolic periods do not allow the formation of an effective counterpulsation wave. Therefore, in situations of extreme hemodynamic decompensation, the use of an left ventricular assisting device or a total artificial heart is inescapable [17–20]. In addition, the ETPACD requires thoracotomy for implantation and therefore in cases of mild hemodynamic decompensation expected to last for a short period of time, the IABP with the advantage of percutaneous insertion remains the first line option.

## Conclusion

In conclusion, this study demonstrated that both ETPACD and IABP provided benefit of circulatory support in acute heart failure with better effect on hemodynamic parameters provided by ETPACD. The advantages of this device, including low cost, ease of implantation and removal, wide availability of the driving consoles, and high biocompatibility, may suggest that ETPACD a desirable alternative for the long-termmechanical support of heart failure or as a bridge to transplantation or myocardial recovery. Therefore, ETPACD with theoretical larger stroke may become a promising counterpulsation device for treatment of heart failure.

## Abbreviations

ETPACD: Extra-thoracic paraaortic counterpulsation device
IABP: Intraaortic balloon pump
CHF: Congestive heart failure
PACD: Paraaortic counterpulsation device
HR: Heart rate
SAP: Systolic aortic pressure
MADP: Mean aortic diastolic pressure
CVP: Central venous pressure
CO: Cardiac output
ECG: Electrocardiogram
LCAF: Left carotid artery flow
ACT: Activated coagulation time
